# Mussel-inspired polydopamine decorated alginate dialdehyde-gelatin 3D printed scaffolds for bone tissue engineering application

**DOI:** 10.3389/fbioe.2022.940070

**Published:** 2022-08-08

**Authors:** Farnaz Ghorbani, Minjoo Kim, Mahshid Monavari, Behafarid Ghalandari, Aldo R. Boccaccini

**Affiliations:** ^1^ Institute of Biomaterials, Department of Materials Science and Engineering, University of Erlangen-Nuremberg, Erlangen, Germany; ^2^ State Key Laboratory of Oncogenes and Related Genes, Institute for Personalized Medicine, School of Biomedical Engineering, Shanghai Jiao Tong University, Shanghai, China

**Keywords:** scaffold, tissue engineering, extrusion-based 3D printing, polydopamine, alginate dialdehyde-gelatin, bone

## Abstract

This study utilized extrusion-based 3D printing technology to fabricate calcium-cross-linked alginate dialdehyde-gelatin scaffolds for bone regeneration. The surface of polymeric constructs was modified with mussel-derived polydopamine (PDA) in order to induce biomineralization, increase hydrophilicity, and enhance cell interactions. Microscopic observations revealed that the PDA layer homogeneously coated the surface and did not appear to induce any distinct change in the microstructure of the scaffolds. The PDA-functionalized scaffolds were more mechanically stable (compression strength of 0.69 ± 0.02 MPa) and hydrophilic (contact angle of 26) than non-modified scaffolds. PDA-decorated ADA-GEL scaffolds demonstrated greater durability. As result of the 18-days immersion in simulated body fluid solution, the PDA-coated scaffolds showed satisfactory biomineralization. Based on theoretical energy analysis, it was shown that the scaffolds coated with PDA interact spontaneously with osteocalcin and osteomodulin (binding energy values of −35.95 kJ mol^−1^ and −46.39 kJ mol^−1^, respectively), resulting in the formation of a protein layer on the surface, suggesting applications in bone repair. PDA-coated ADA-GEL scaffolds are capable of supporting osteosarcoma MG-63 cell adhesion, viability (140.18% after 7 days), and proliferation. In addition to increased alkaline phosphatase secretion, osteoimage intensity also increased, indicating that the scaffolds could potentially induce bone regeneration. As a consequence, the present results confirm that 3D printed PDA-coated scaffolds constitute an intriguing novel approach for bone tissue engineering.

## 1 Introduction

Bones are load-bearing structures that serve as mechanical support for the body and provide protection for the organs. Bone is well-known for its ability to self-repair, where it undergoes remodeling, maturation, differentiation, and resorption throughout one’s life to maintain healthy bone (Zhang et al., 2022). Defects in bones beyond the capacity for self-repair occur as a result of accidents, aging, infection, cancer, surgical resection, and may require surgical intervention, resulting in the need for appropriate clinical treatment. In spite of the availability of alternatives, such as autografts and allografts, the high cost, immune rejections, limited number of donors, the requirement of multiple surgeries, and the potential for viral transmission limit the use of such transplants (Rupp et al., 2022). Therefore, the development of biological repair strategies, including tissue engineering (TE), focusing on the synthesis and functionalization of biomaterials for bone regeneration is receiving increasing attention (Li et al., 2021; Hao et al., 2022; Wang et al., 2022).

Bone TE is used to replace, restore, or accelerate the regeneration of damaged bone tissue through the interaction between cells and the artificial construct called scaffold. Apart from the traditional methods for fabricating scaffolds such as electrospinning, freeze-drying, solvent casting, and others, additive manufacturing and rapid prototyping strategies have become very popular (Qu et al., 2021). There are a number of 3D printing technologies available, including stereolithography (SLA), fused deposition modeling (FDM), selective laser sintering (SLS), and extrusion-based 3D printing (Xu et al., 2022). One of the most popular methods of 3D printing is extrusion-based technology, which is able to print complex and hierarchical constructs for patient-specific applications (Bandyopadhyay et al., 2021; Beheshtizadeh et al., 2021).

Hydrogels have already been extensively studied for bone regeneration applications due to their biocompatibility, biodegradability, printability, and most importantly, their ability to mimic the microenvironment of natural tissues (Xue et al., 2022). They are well known for stimulating cell attachment, proliferation, and growth, as well as supporting the regeneration of native extracellular matrix (ECM) structures (Kreller et al., 2021). Alginate has been extensively studied as a potential component of 3D printing inks (Reakasame et al., 2021). It is an appealing candidate for TE applications due to its biocompatibility, low toxicity, printability, injectability, mild processing conditions, and low cost. Despite this, alginate does not possess the essential characteristics necessary for TE, such as no binding sites for cell attachment, and in the case of bone TE, pure alginate lacks mineralization capability and mechanical strength (Genes et al., 2004). Gelatin is a biocompatible biopolymer that has a similar molecular structure and function to collagen, and it is used extensively in tissue engineering and cell culture experiments for its ability to provide powerful biological and chemical signals that allow for the replication of many different types of cells. In spite of gelatin’s advantages and wide range of applications, there are a few drawbacks, including its poor mechanical properties and fast degradability (Afewerki et al., 2019; Bello et al., 2020). To compensate for the mentioned drawbacks, the use of alginate dialdehyde-gelatin (ADA-GEL) has already been investigated for bone TE (Sarker et al., 2016; Monavari et al., 2021), cartilage TE (Balakrishnan et al., 2014; Kreller et al., 2021), wound dressings (Balakrishnan et al., 2005), as well as bioprinting of artificial cancer models (Bednarzig et al., 2021). ADA is caused by the partial oxidation of alginate and exhibits cleavage of vicinal glycols in alginate that negatively impact its ability to absorb water (O’Brien et al., 2005). Additionally, the crosslinking ability of ADA with calcium ions will be reduced when the oxidation exceeds 10%, resulting in less mechanical properties than alginate (Gomez et al., 2007). Consequently, ADA-GEL offers promising prospects in terms of physicochemical, biocompatibility and cellular interaction.

The ability to generate hydroxyapatite (HA) is essential for the bone regeneration process (Tang G. et al., 2021). In this regard, the surface modification of ADA-GEL scaffolds with bioactive components is a promising method to improve the mineralization potential of ADA-GEL. Recently, mussel-inspired polydopamine (PDA), containing rich catechol and amine content, has been studied for use as a coating for biomedical implants due to its hydrophilicity, low toxicity to pre-osteoblast cells, and ability to facilitate and promote crystallization of HA by providing more active sites (Tang Y. et al., 2021; Feng et al., 2021). According to recent studies, PDA can also enhance scaffold mechanical stability, induce angiogenesis and osteogenesis, and enhance the integrability of implants with the surrounding tissue (Sun et al., 2021; Yu et al., 2021).

We conducted this study to investigate the effect of bio-inspired PDA functionalization on extrusion-based 3D printed ADA-GEL scaffolds for bone regeneration. In this study, the performance of the PDA-coated ADA-GEL scaffolds was evaluated *in-vitro* to determine the impact of PDA coatings on mechanical strength, hydrophilicity, degradation, and biomineralization. Furthermore, computational studies were conducted to gain insights on the possible scaffold interactions with bone-related model proteins. The cell-scaffold interaction was also assessed in an effort to determine the cell adhesion, proliferation, and osteogenic properties of the polymeric scaffold, especially after PDA decoration.

## 2 Materials and methods

### 2.1 Materials

Gelatin from porcine skin (gel strength ∼300 g Bloom, Type A), sodium alginate (sodium salt of alginic acid from brown algae, suitable for immobilisation of micro-organisms, guluronic acid concent 65–70%), dopamine hydrochloride (Mw = 189.64 g/mol), 2-propanol (Mw = 50.10 g/mol), hydrochloric acid (Mw = 36.46 g/mol), sodium (meta) periodate (Mw = 213.89 g/mol), sodium chloride (Mw = 58.44 g/mol), potassium phosphate dibasic trihydrate (Mw = 228.22 g/mol), Bradford reagent (for 0.1–1.4 mg/ml protein), and penicilin-streptomycin (suitable for cell culutre, lyophilised) were purchased from Sigma Co., Germany. Calciumchloride dihydrate was purchased from Bernd Kraft Co., Germany. TRIS (Mw = 121.14 g/mol) was purchased from ROTH Co., Germany. Magnesium chloride hexahydrate (Mw = 203.30 g/mol) was purchased from Honeywell Fluka Co., Germany. Potassium chloride (Mw = 74.55 g/mol), sodium hydrogen carbonate (Mw = 84.01 g/mol), ethanol (Mw = 46.07 g/mol), glutaraldehyde (25% aqueous solution, Mw = 100.11 g/mol), and formaldehyde (Mw = 30.031 g/mol) were purchased from Merck Co., Germany. Calcein-AM was purchased from Invitrogen, United States DMEM (Dulbecco’s Modified Eagle Medium) (1X), trypan blue stain (0.4%), DPBS (Dulbecco’s Phosphate Buffered Saline, 1X), and HBSS (Hank’s Balanced Salt Solution) were purchased from Gibco Co., Germany. Ethylene glycol (Mw = 62.07 g/mol) and sodium sulphate anhydrous (Mw = 142.02 g/mol) were purchased from VWR International Co., Germany. Osteoimage™ mineralization assay was purchased from Lonza, United States of America.

### 2.2 Preparation of ADA-GEL 3D printed scaffolds

ADA-GEL was formulated using a combination of alginate dialdehyde (ADA) and gelatin (GEL) which were covalently cross-linked, as investigated in our previous study (Monavari et al., 2021). The ADA-GEL synthesis is here briefly described. Following dissolving sodium alginate in ethanol (0.2 g/ml), 0.125 M sodium metaperiodate solution was added in the same volumetric amount to oxidize the hydroxyl groups in alginate. After 6 hours of keeping the mixture solution in the dark at room temperature, ethylene glycol (10%v/v) was added in order to stop oxidation and further stored for 30 min. The suspension was dialyzed for 5 days using a semipermeable dialysis membrane (MWCO 6–8 kDa, Spectrum Lab, USA) against ultrapure water (Direct-Q^®^, Merck, Germany) to eliminate metaperiodate ions. In order to obtain dry ADA, lyophilization was carried out using a freeze dryer (Alpha 1-4 LSCplus, Christ, Germany).

ADA solution with a concentration of 2.5 wt% was prepared by dissolving in PBS and stirring it at room temperature overnight. The GEL-PBS solution (3.75 wt%) was added gradually to the ADA solution to obtain a printable ink. ADA-GEL scaffolds were fabricated using a pneumatic extrusion-based 3D printer (typeBioScaffolder 3.1, GeSiM, Großerkmassnadorf, Germany). The scaffold was designed using the software included with the 3D printer CAGD (Computer-Aided Geometric Design). A square grid-like 3D scaffold, 10 mm in length, was designed to be printed in four layers. The printer cartridge containing ADA-GEL was connected to a compressed air supply, and the hydrogel was printed layer by layer using a 25 G nozzle. Following the printing of the scaffolds, 0.6 M CaCl_2_ was used to cross-link the constructs, which were then washed with HBSS to remove unreacted CaCl_2_. ADA-GEL scaffolds that would be assessed without a PDA coating, as control group, were lyophilized for 3 days.

### 2.3 PDA decoration on ADA-GEL scaffolds

Functionalization of the scaffolds produced using 3D printing was accomplished according to our previous investigation (Ghorbani et al., 2019b). This is achieved by mixing ultrapure water and 2-propanol in a volumetric ratio of 5:2, followed by the addition of TRIS (10 mM). The pH was adjusted to 8.6 by adding HCl (1 M) dropwise under stirring conditions. Subsequently, 2 mg/ml of dopamine hydrochloride solution was prepared and was gradually added to the main solution while stirring. After embedding the scaffolds manually in the prepared medium, they were incubated in a thermoshaker (IKA KS 4000 I Control) at 37°C in the darkness. After 24 h, the scaffolds were rinsed with ultrapure water and lyophilized for 3 days.

### 2.4 Characterization

Field emission scanning electron microscopy (FE-SEM, Auriga, Carl-Zeiss, Germany) and light microscopy (Stemi 508, Carl-Zeiss, Germany) were used to observe the morphology of the lyophilized scaffolds. Light microscope images were also used to quantify the pore size and strand diameter using image measurement software (ImageJ).

The chemical bonding structure of lyophilized non-modified ADA-GEL scaffolds and PDA-coated ADA-GEL scaffolds was studied using a Fourier-transform infrared spectrometer (IRAffinity-1S, Shimadzu, Japan) with 40 scans in the range 400–4,000 cm^−1^.

A standard uniaxial mechanical tester (5967, Instron, United States) was used to evaluate the compressive strength of lyophilized scaffolds through a 100 N load cell and a crosshead speed of 0.5 mm/min.

In order to study the hydrophilicity, a drop shape analyzer (DSA30 Expert, Kruss, Germany) was used to measure the water drop contact angle.

To determine the scaffolds’ water absorption capacity, ADA-GEL and PDA-coated ADA-GEL scaffolds were immersed in 10 ml of MEM alpha cell medium (1 µL of gentamicin was added to each mL of medium) in a six-well tissue culture plate. ADA-GEL and PDA-coated ADA-GEL scaffolds were weighed at in dry state and after 2, 4, 6, 24, and 96 h of incubation in a thermoshaker at 37 ± 0.5 °C in dark condition. The absorption ratio was calculated for the ADA-GEL and PDA-coated ADA-GEL scaffolds according to Eq. 1 until maximum fluid absorption occurred (Nokoorani et al., 2021):

$$\mathrm{Swelling ratio}\left(\%\right)=[(\mathrm{W-}W_{i}/W_{i}]\mathrm{*100}$$ (1)

where W_i_ is the dry weight of the scaffold and W is its maximum equilibrated swollen weight.

*In-vitro* biodegradation behavior of the scaffolds was assessed by immersion of the ADA-GEL and PDA-coated ADA-GEL scaffolds in 10 ml of MEM alpha cell medium (1 µL of gentamicin was added to each mL of medium) in a six-well tissue culture plate. The ADA-GEL and PDA-coated ADA-GEL scaffolds were weighed at days 4, 6, 8, 12, 15, and 18, while the medium was actively refreshed at each time interval. The scaffolds were washed with ultrapure water and lyophilized following 18 days of incubation at 37 ± 0.5 °C in order to characterize them using FE-SEM, light microscopy, and FTIR. The degradation ratio of the scaffolds was calculated based on Eq. 2 (Montes et al., 2022):

$$\mathrm{Biodegradation ratio}\left(\%\right)=\left|\left[\left(W_{w}-W_{i}\right)/W_{i}\right]\right|\mathrm{*100}$$ (2)

where the initial weight (W_i_) is the weight of the ADA-GEL scaffold at its maximum equilibrated swollen weight, and W_w_ is the wet weight of the scaffolds at predetermined time interval.

To evaluate the acellular bioactivity of the PDA-coated ADA-GEL scaffold for osteoregeneration, an *in-vitro* bioactivity study was performed. To conduct the bioactivity test, simulated body fluid (SBF) solution was prepared (Kokubo and Takadama, 2006) and scaffolds (10 * 10 * 1 mm^3^) were immersed in 10 ml of SBF solution and incubated at 37 ± 0.5 °C in a thermoshaker under dark conditions. The SBF solution was refreshed every other day. Following 18 days incubation, lyophilized scaffolds were characterized by FE-SEM, light microscopy, FTIR, and XRD analyses. The X-ray diffractometer (MiniFlex 600, Rigaku, Japan) records the spectrum in the range of 2θ angles 20–80° using Cu-Kα (λ 1.5418°A) radiation.

### 2.5 Molecular docking simulation

PDA coating layer interactions with osteocalcin and osteomodulin have been studied using AutoDock Vina (Trott and Olson, 2010). Protein structures were obtained from the AlphaFold Protein Structure Database. According to previous works (Ghorbani et al., 2021a; Ghalandari et al., 2021), the repeated-basic unit of a PDA molecule was modeled and used for docking calculations. In accordance with the classical preparation instructions (Abbasi-Tajarag et al., 2016; Gholamian et al., 2017; Ghorbani et al., 2021b) docking calculations were performed. Preparation of input files and data analysis were carried out by the AutoDock Tools 1.5.4. (Morris et al., 2009). and the VMD package (Humphrey et al., 1996).

### 2.6 Cell-scaffolds interactions

A series of *in-vitro* experiments was carried out using MG-63 cells. Scaffolds were seeded with 50,000 cells/ml in DMEM supplemented with 10% FBS and 100 U/ml penicillin-streptomycin and maintained at 37 ± 0.5 °C, 5% CO_2_, and 95% humidity for 2 days to study cell attachment. The cultured cells on the scaffolds were then fixed with glutaraldehyde and formaldehyde-containing solutions, washed with HBSS, dehydrated with ethanol series, and air-dried for FE-SEM analysis.

Calcein-AM staining assay was performed to determine cell viability at days 2, 4, and 7. Therefore, the scaffolds seeded with cells were incubated in 4 μM calcein-AM/1 ml HBSS at 37 ± 0.5 °C, 5% CO_2_, and 95% humidity for 1 h. The constructs were then washed in sterile HBSS before being fixed in fluorescent fix solution and cell viability was determined using a fluorescence microscope (Axio Observer, Carl-Zeiss, Germany).

WST-8 assay was used to evaluate the proliferation of the cultured cells on scaffolds after 2, 4, and 7 days. The cell-cultured samples were incubated in DMEM containing 3% WST-8 for 3 h at 37°C, 5% CO_2_, and 95% humidity after removing the medium at each time point. In the next step, 100 µl of the medium was separated to measure the optical density at 450 nm using a microplate reader (FLUOstar Omega, BMG LABTECH, Germany).

ALP activity was measured in order to evaluate the osteoblastic behavior of cultured cells. Cells were lysed with lysis buffer and the medium was centrifuged at 1,200 rpm for 10 min after 7 and 21 days of cell culture. ALP-mix solution containing p-NPP was added to the supernatant and incubated for 180 min. The reaction was stopped with the addition of NaOH. Analyzing absorbance at wavelengths of 405 and 690 nm enabled the measurement of ALP activity. As a final step, ALP levels were adjusted for total protein content determined by the Bradford assay. To do so, the supernatant of the lysed cells (25 µl) was transferred to a cuvette containing the Bradford protein assay kit (AppliChem GmbH, Germany) (975 µl). After 10 minutes of incubation, the optical absorbance of the as-prepared solution was determined via UV-Vis spectrophotometry at 595 nm in dark conditions.

To assess the hydroxyapatite content of bone-like nodules deposited by cells, a fluorescent osteoimage mineralization assay was performed. The experiment was conducted after 21 days in accordance with the Lonza kit protocol. The cells were fixed after removal of the medium and washing. The cultured cells were first washed two times with wash buffer solution. After staining the cells in dark condition and incubating them for 30 min, they were washed three times and monitored for mineralization using a fluorescence microscope and microplate reader.

### 2.7 Statistical analysis

Each experiment was repeated five times (*n* = 5) and data were reported as the mean ± standard deviation. The significance of the average values was calculated using a *t*-test calculator; *p* ≤ 0.05 was considered significant.

## 3 Results and discussion

### 3.1 Microstructural observation

**FIGURE 1:**
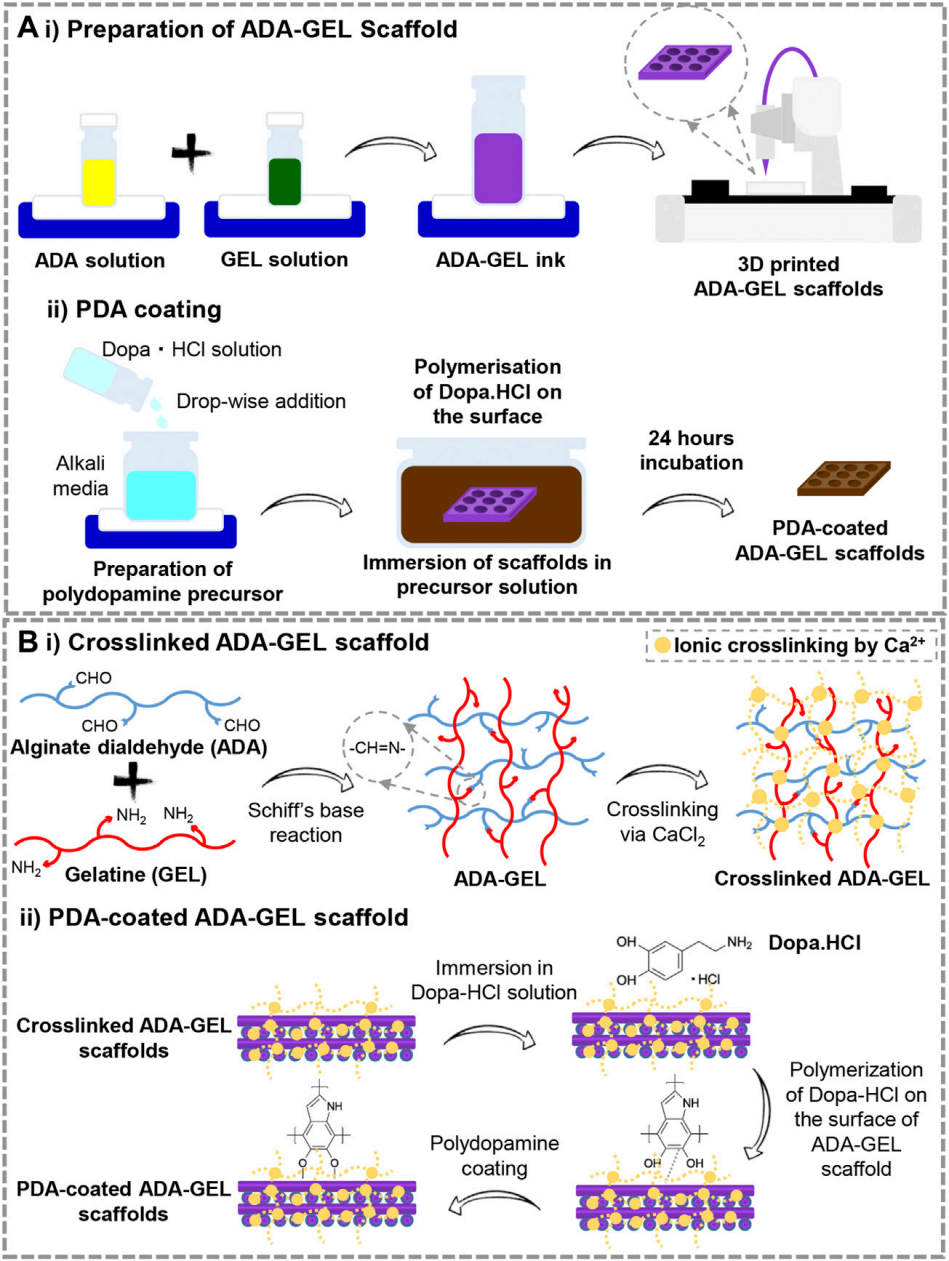
**(A)** Schematic illustration of the preparation steps of 1) 3D printed ADA-GEL scaffolds and 2) PDA decoration of the printed ADA-GEL scaffolds. **(B)** Schematic illustration of the process of 10 cross-linking and 2) PDA functionalizing ADA-GEL scaffolds.

**FIGURE 2:**
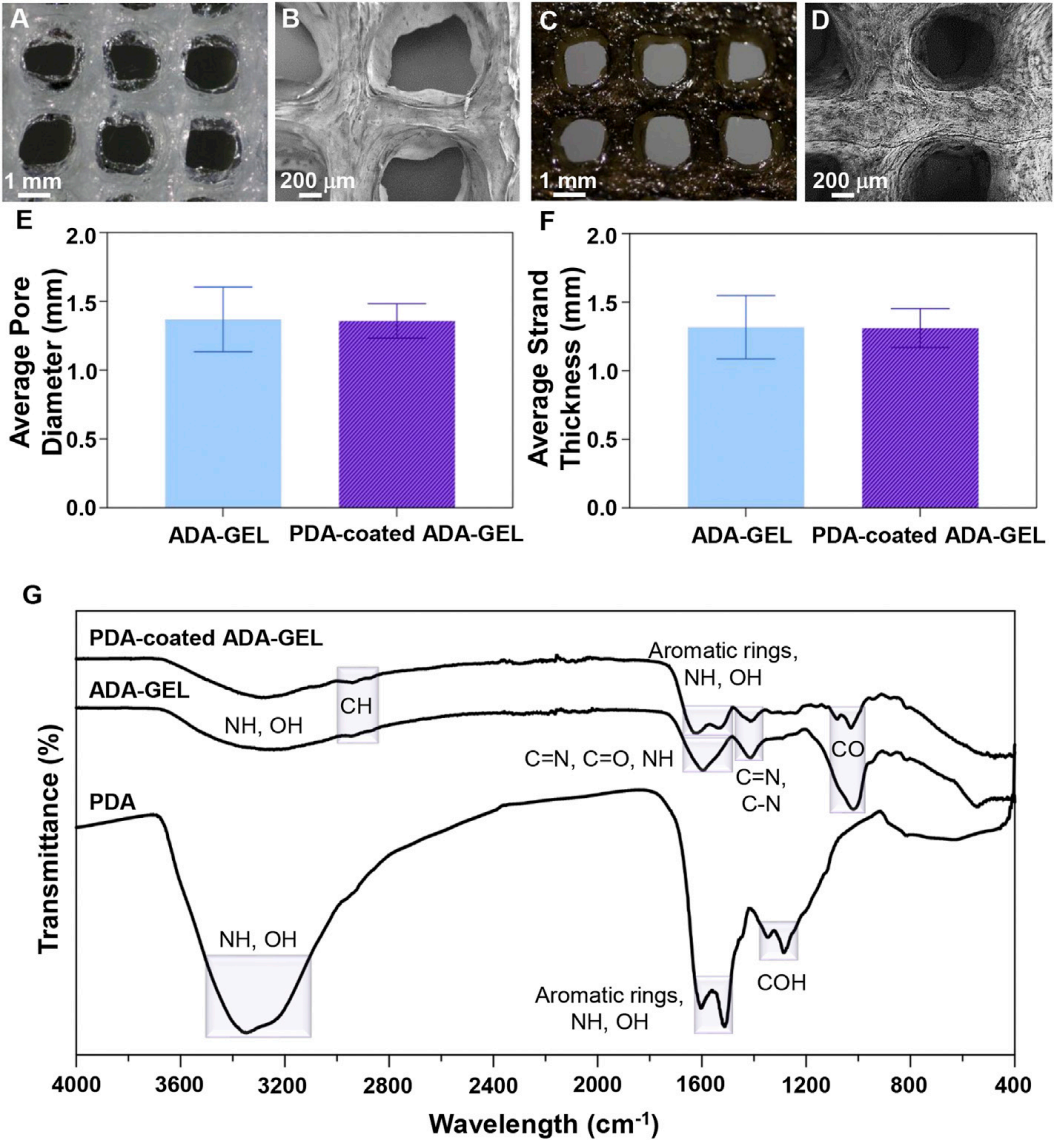
Microstructural and chemical evaluation of scaffolds. **(A,C)** Light microscope images and **(B,D)** FE-SEM images of **(A,B)** ADA-GEL and **(C,D)** PDA-coated ADA-GEL scaffolds. **(E)** Average pore diameter and **(F)** average strand thickness of ADA-GEL and PDA-coated ADA-GEL scaffolds. **(G)** Chemical characterization of polymeric scaffolds. FTIR spectrum of PDA, ADA-GEL, and PDA-coated ADA-GEL scaffold.

Comparatively to conventional scaffold fabrication technologies, 3D printing provides an accurate and reproducible scaffold fabrication technique that permits the development of personalized and hierarchical scaffold models tailored to the needs of the target tissue. Figure 1A presents a schematic illustration of the ADA-GEL ink preparation for 3D printing along with the procedure for PDA coating on the scaffolds. A previous published study of our group (Distler et al., 2020) has shown that ADA is oxidized by 13%. Here, the degree of oxidation (DO) of ADA was estimated by the determination of the difference between the initial and present amount of IO_4_ ions before quenching the reaction with ethylene glycol using the method. Our study applied a similar procedure by using alginate and sodium periodate. Figures 2A–D displays the morphology of the lyophilized ADA-GEL and PDA-coated ADA-GEL scaffolds are determined by FE-SEM and light microscopy. As a consequence of this study, ADA-GEL scaffolds were fabricated with a complex porous structure with spatially adjustable geometry and microstructure (Moore et al., 2020), which can improve nutrient absorption and promote cell adhesion, proliferation, and differentiation (Yan et al., 2005). 3D printed scaffolds were then modified by polymerizing a layer of PDA on the surface. It was found that both experimental groups formed an interconnected microtubule-like microstructure with slight contraction in comparison to the 3D designed file. Sphere-shaped PDA was also deposited homogeneously and uniformly on the surface of the ADA-GEL scaffolds, demonstrating self-oxidation of dopamine hydrochloride without adversely affecting the 3D structural integrity and architecture of the construct. According to the average pore diameter (Figure 2E), the pore sizes for ADA-GEL and PDA-coated ADA-GEL scaffolds were 1.37 ± 0.23 mm and 1.37 ± 0.12 mm, respectively. Similarly, the average strand thickness (Figure 2F) was 1.32 ± 0.23 mm for ADA-GEL constructs and 1.31 ± 0.14 mm for PDA-coated ADA-GEL ones. As reported in a similar study, surface modification *via* PDA can improve bioactivity and cell behavior without any adverse effect on the pore structure (Jo et al., 2013).

### 3.2 Chemical characterization

In order to determine the chemical interactions of PDA with ADA-GEL, as well as to examine the successful cross-linking of samples, FTIR spectra of raw materials and composite structures were examined. Figure 2G depicts the FTIR spectra of PDA, ADA-GEL and PDA-coated ADA-GEL. For ADA-GEL, the peak around 2,933 cm^−1^ corresponds to CH. Besides, the observed peak at 1,599 cm^−1^ correspond to C=N, C=O, and NH bonds. C=N and C-N characteristic peak was observed at 1,414 cm^−1^. The peak at 1,015 cm^−1^ is caused by CO bonds. Broad peaks were observed at about 3,200–3,500 cm^−1^ belonging to amine and hydroxyl groups in both PDA and ADA-GEL. Peaks are observed around 1,603 cm^−1^ and 1,513 cm^−1^ for aromatic rings, correlated with the OH and NH groups in the PDA chemical structure. Additionally, PDA COH bonds were identified at 1,345 and 1,284 cm^−1^ (Ghorbani et al., 2019b). The presence of PDA peaks on PDA-coated ADA-GEL scaffolds confirms the spontaneous polymerization of dopamine hydrochloride on the ADA-GEL substrates.

Figure 1B illustrates the preliminary chemical reaction that leads to ADA-GEL formation and the cross-linking of ADA-GEL by Ca^2+^ ions. Under Schiff’s base reaction, alginate dialdehyde interacts with gelatin (O’Brien et al., 2005; Boanini et al., 2010). ADA-GEL scaffolds undergo ionic gelation when Ca^2+^ ions bound to G-blocks in alginate (Reakasame et al., 2021). The reaction of ADA with GEL goes through amidation coupling. Here, an excessive relative amount of gelatin to ADA needs to be selected in order to improve cell interactions, ensure all carboxyl groups of ADA reacted with the amine group of gelatin, decrease the gelation time to 10 min, achieve neutralization of acidity of ADA solution, obtain higher thermal stability at lower temperatures, and control remained uncrosslinked gelatin in high concentration. Considering these issues, ADA (2.5%) –GEL (3.75%) was selected as the optimum ratio, similar to the early Sarkar et al. (Sarker et al., 2014) investigation. Further functionalization of cross-linked ADA-GEL should be accomplished with PDA. Surface decoration with PDA can be affected by the catechol moiety of PDA’s chemical structure. Nielsen et al. (2013) study indicates that the polymerization of catechol increases binding forces, which promotes chemical reaction initiation. Considering all the above, C-C binding happens between neutralized dopamine hydrochloride and open-chain oligomers that are contained within the structure of the PDA. There can, therefore, be a possibility for specific kinds of interaction to take place in PDA (Liebscher et al., 2013). Generally, neutralization of dopamine hydrochloride and its spontaneous oxidation under alkaline conditions generates polydopamine. As a consequence, dopaminequinone is formed, which is followed by a 1,4- Michael addition reaction. The course of the oxidation process will lead to the transformation of leucodopaminechrome into dopaminechrome and a subsequent rearrangement to 5,6-dihydroxyindole. Finally, PDA can be synthesized from the o-quinone of the 5,6-dihydroxyindole in conjunction with catechol groups (Wei et al., 2010; Yu et al., 2010). Tang et al. (2021a) reported that attachment of PDA to substrates happens via covalent binding (Michael-type addition, Schiff base reactions) or noncovalent binding (hydrogen bonding, π−π stacking, metal chelating.

### 3.3 Mechanical behavior

**FIGURE 3:**
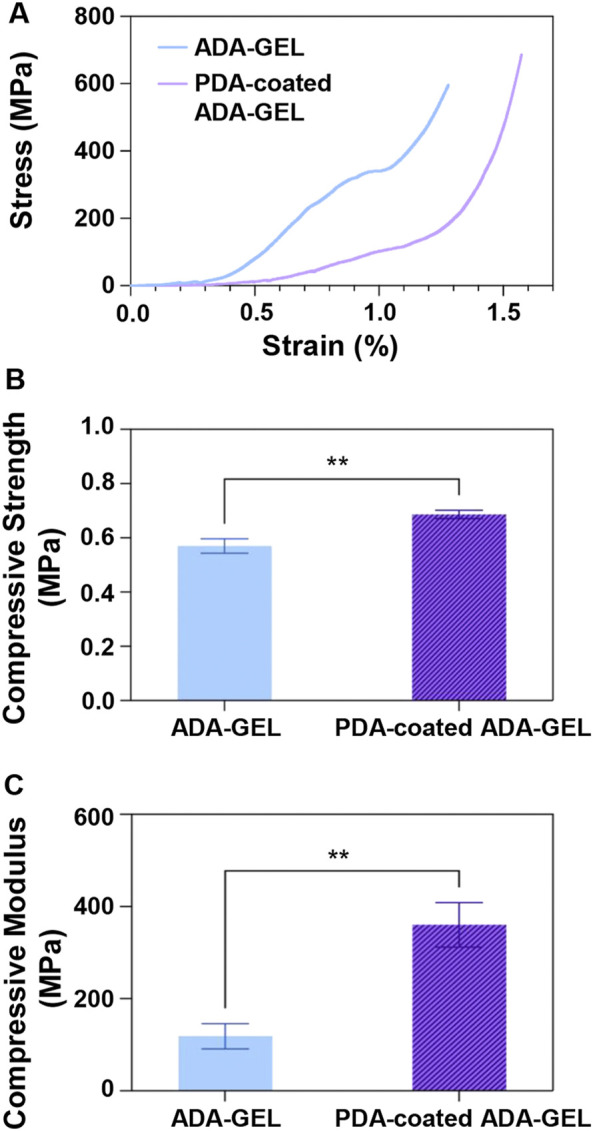
Mechanical behavior of the ADA-GEL and PDA-coated ADA-GEL scaffolds. **(A)** Stress-strain curve, **(B)** compressive strength, and **(C)** compressive modulus of scaffolds. Differences are considered very statistically significant (**p < 0.01).

Mechanical properties are crucial for bone TE since the scaffolds must exhibit the initial load-bearing ability to support bone regeneration. A clear relationship exists between mechanical stability of the scaffolds and cellular activity. In this regard, mitogen-activated protein kinases are influenced by the elastic modulus and help the regulation of osteogenic differentiation through actin-cytoskeleton (Khatiwala et al., 2007). Therefore, uniaxial compression tests (Figure 3) were carried out to evaluate the mechanical properties and performance of lyophilized ADA-GEL and PDA-coated ADA-GEL scaffolds. Figure 3A shows the stress-strain diagrams of both ADA-GEL and PDA-coated ADA-GEL scaffolds, showing that both compositions exhibit a low and narrow elastic regime. ADA-GEL coated with PDA appears to have a more uniform deformation pattern in comparison to ADA-GEL. Toughness values were 247.57 ± 0.03 and 171.65 ± 0.01 J/mm^3^ for ADA-GEL scaffolds and PDA-coated ADA-GEL ones, respectively. Accordingly, ADA-GEL scaffolds showed higher toughness, since a large amount of energy needs to be absorbed in order to deform plastically and fracture the scaffolds.

Further, as illustrated in Figure 3B, the PDA-coated ADA-GEL scaffold showed a compressive strength of 0.69 ± 0.02 MPa, which was approximately 20% higher than the non-coated ADA-GEL scaffold of 0.57 ± 0.03 MPa. The PDA coating on the ADA-GEL scaffolds, as shown in Figure 3C, exhibits the necessary modulus compared with natural cancellous bone (elastic modulus in the range of 10–2,000 MPa (Ostrowska et al., 2014)) and has significantly improved compressive modulus (360 ± 48 MPa) in comparison to the compressive modulus of ADA-GEL (118 ± 27 MPa). These findings suggest that the PDA coating enhances ADA-GEL scaffolds’ mechanical and structural integrity. A considerable benefit of the PDA coating layer is achieved by the strands’ void space infiltration and formation of a denser structure. Also, the PDA layer acts as a glue for improving interconnectedness and adhesion of the layers (Kim et al., 2018). Interestingly, Huang et al. (Huang et al., 2014) indicated that prolong coating time would not show any improvement in the mechanical strength of scaffolds. PDA coating was applied on scaffolds and both the Young’s modulus and compressive strength were improved in a similar study (Xie et al., 2012). It has been demonstrated by Shin et al. (Shin et al., 2011) that the modulus of elasticity of PCL and PLA copolymers increased upon application of PDA, which was caused by the polymerization of PDA on the polymers’ surfaces.

### 3.4 Absorption capacity and biodegradation

**FIGURE 4:**
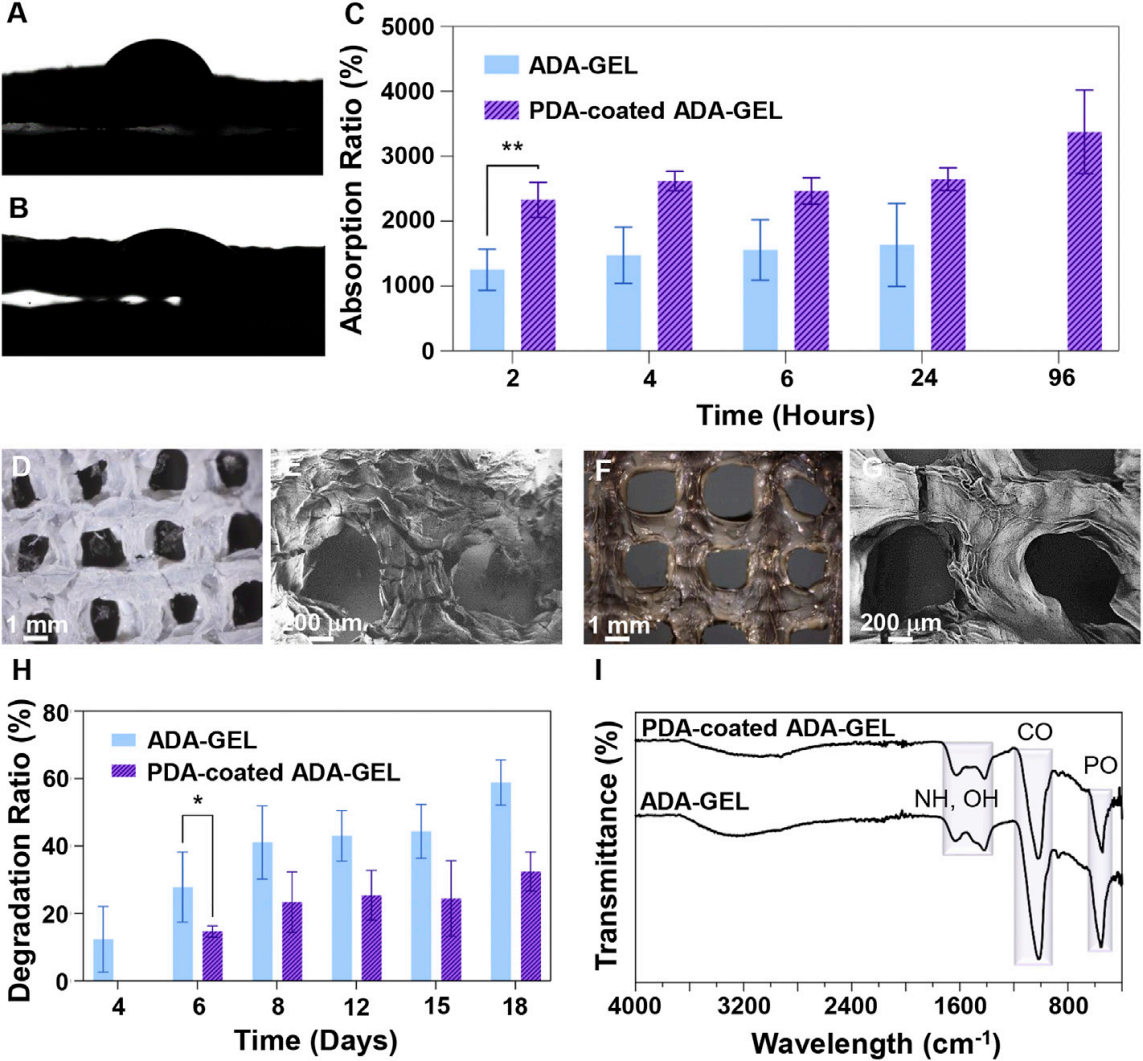
Interaction of scaffolds with biological-like fluids. **(A,B)** Contact angle measurements of **(A)** ADA-GEL and **(B)** PDA-coated ADA-GEL scaffolds. **(C)** Absorption ratio of ADA-GEL and PDA-coated ADA-GEL scaffolds over time (2, 4, 6, 24, and 96 h). **(D,F)** Light microscope images and **(E,G)** FE-SEM image of **(D,E)** ADA-GEL and **(F,G)** PDA-coated ADA-GEL scaffolds after a 18-days immersion in medium. **(H)** Degradation ratio of ADA-GEL and PDA-coated ADA-GEL scaffolds over time (4, 6, 8, 12, 15, and 18 days). **(I)** FTIR spectrum of ADA-GEL and PDA-coated ADA-GEL after a 18-days degradation test. Differences are considered statistically significant (*p ≤ 0.05) and very statistically significant (**p < 0.01).

A crucial parameter that determines cell-scaffold interactions is the water-scaffold interface and scaffold wettability, which can be measured by performing a water droplet contact angle test. The contact angle (Figures 4A,B) of 58 was obtained for ADA-GEL while 26 was measured for PDA-coated ADA-GEL. In accordance with the results of the contact angle test, the PDA-coated ADA-GEL hydrogel exhibits increased hydrophilicity relative to the uncoated ADA-GEL hydrogel. A significant improvement is anticipated in the hydrophilicity of PDA as a result of the abundance of hydrophilic functional groups in amine and catechol groups. PDA coating has been shown to reduce the contact angle of PCL and by boosting dopamine concentrations this effect was further intensified (Cheng et al., 2016).

The absorption ratio over time is shown in Figure 4C. Absorption ratios of ADA-GEL were 1,254 ± 316%, 1,477 ± 431%, 1,558 ± 466%, and 1,635 ± 639% at 2, 4, 6, and 24 h of incubation, respectively. A rapid amount of fluid is absorbed at the beginning followed by a slow, steady rate of absorption until the maximum amount of fluid has been absorbed. For the PDA-coated ADA-GEL, the absorption ratio was 2,330 ± 272%, 2,621 ± 149%, 2,467 ± 200%, 2,648 ± 173%, and 3,377 ± 644% at 2, 4, 6, 24, and 96 h of incubation, respectively. In total, ADA-GEL hydrogels coated with PDA had an absorption ratio almost twice as high as ADA-GEL hydrogels without coating. This result indicates that PDA-coated ADA-GEL has superior fluid intake capabilities over uncoated ADA-GEL. Also, whereas ADA-GEL hydrogels reached their maximum fluid uptake by 24 h of incubation, PDA-coated ADA-GEL hydrogels demonstrated an increased stability in fluid absorption up to 96 h, supporting the conclusion about the increased stability (chemical, mechanical) and structural integrity caused by PDA coating. As demonstrated in a similar investigation, PDA coating on thermoplastic polyurethane is capable of improving water uptake and cellular interaction (Lin et al., 2020).

As with hydrophilicity and absorption ratio, the chemical composition and microstructure play a role in the degradation behaviour of biomaterials. (Figures 4D–G) depicts light microscope and FE-SEM images of ADA-GEL and PDA-coated ADA-GEL scaffolds after the biodegradation test. As a result of the subsequent assessment of the morphology and mechanical properties of the PDA-coated ADA-GEL, it has been shown that the coating acts as a very thin barrier to give the scaffold structural integrity. These observations are corroborated by the light microscope and FE-SEM images, where PDA-coated ADA-GEL scaffolds show fewer cracks and peeling on the surface. Therefore, the PDA-coated ADA-GEL scaffolds are more likely to withstand mechanical loads during the bone regeneration process than those without PDA coating.

A scaffold designed for tissue regeneration should demonstrate a synchronized degradation rate with that of the regeneration process, as well as remain stable throughout the period of tissue regeneration. In typical bone injury cases, the repair process begins within the first 2 weeks before the start of the remodelling phase. Regeneration is complete approximately 4 to 7 weeks later. Figure 4H illustrates the degradation ratio relative to weight for hydrogels after 18 days in cell culture medium. It was noted that ADA-GEL began to degrade after 24 h in cell culture medium, and the degradation ratio was 12 ± 9%, 28 ± 10%, 41 ± 10%, 43 ± 7%, 44 ± 8%, and 59 ± 7% at 4, 6, 8, 12, 15, and 18 days of incubation, respectively. A steep increase in degradation ratios is perceived at 4to 6 days, followed by a stable increase at 8, 12, and 15 days, with a slight jump of degradation ratios at 18 days after incubation. ADA-GEL coated with PDA shows a similar degradation pattern but with a slower degradation rate. ADA-GEL decorated with PDA was degraded at a rate of 15 ± 2%, 23 ± 9%, 25 ± 7%, 24 ± 11%, and 32 ± 6% after 6, 8, 12, 15, and 18 days of incubation, respectively. Recently the degradation of ADA with various DO has been investigated (Distler et al., 2020). Briefly, different degrees of oxidation by utilizing different amounts of ∼NaIO_4_ oxidant (6.25 and 9.375 mmol; DO:13% and 19%) were investigated, hypothesizing that changing the oxidation degree can alter printed scaffold degradation. A slower initial degradation behavior was observed for ADA with a lower oxidation degree (nNaIO_4_: 6.25 mmol, DO: 13*%*)*.* It must be considered that degradation of ADA-containing substrates leads to polymer chain incision as well as fragmentation and molecular weight reduction. The reduction in the molecular weight increases degradation and reduces the scaffold mechanical stability (Shin et al., 2011). In our study, the degradation test results indicate that the PDA-coated ADA-GEL remains structurally durable and does not degrade extensively.

Furthermore, we evaluated the chemical properties of ADA-GEL and PDA-coated ADA-GEL scaffolds by using FTIR in order to better understand how the scaffolds degrade (Figure 4I). In contrast to the peak intensities of ADA-GEL and PDA-coated ADA-GEL before biodegradation, the peak intensity at 1,599 cm^−1^, which is associated with C=N, C=O, and NH bonds, is reduced in both ADA-GEL and PDA-coated ADA-GEL scaffolds after 18 days in cell culture medium. Additionally, the peaks at 1,513 cm^−1^ corresponding to aromatic rings, OH, and NH groups in the PDA-coated ADA-GEL disappeared. It is evident that a substantial amount of amine and hydroxyl groups have been lost due to biodegradation in cell culture medium for 18 days.

### 3.5 Bioactivity

**FIGURE 5:**
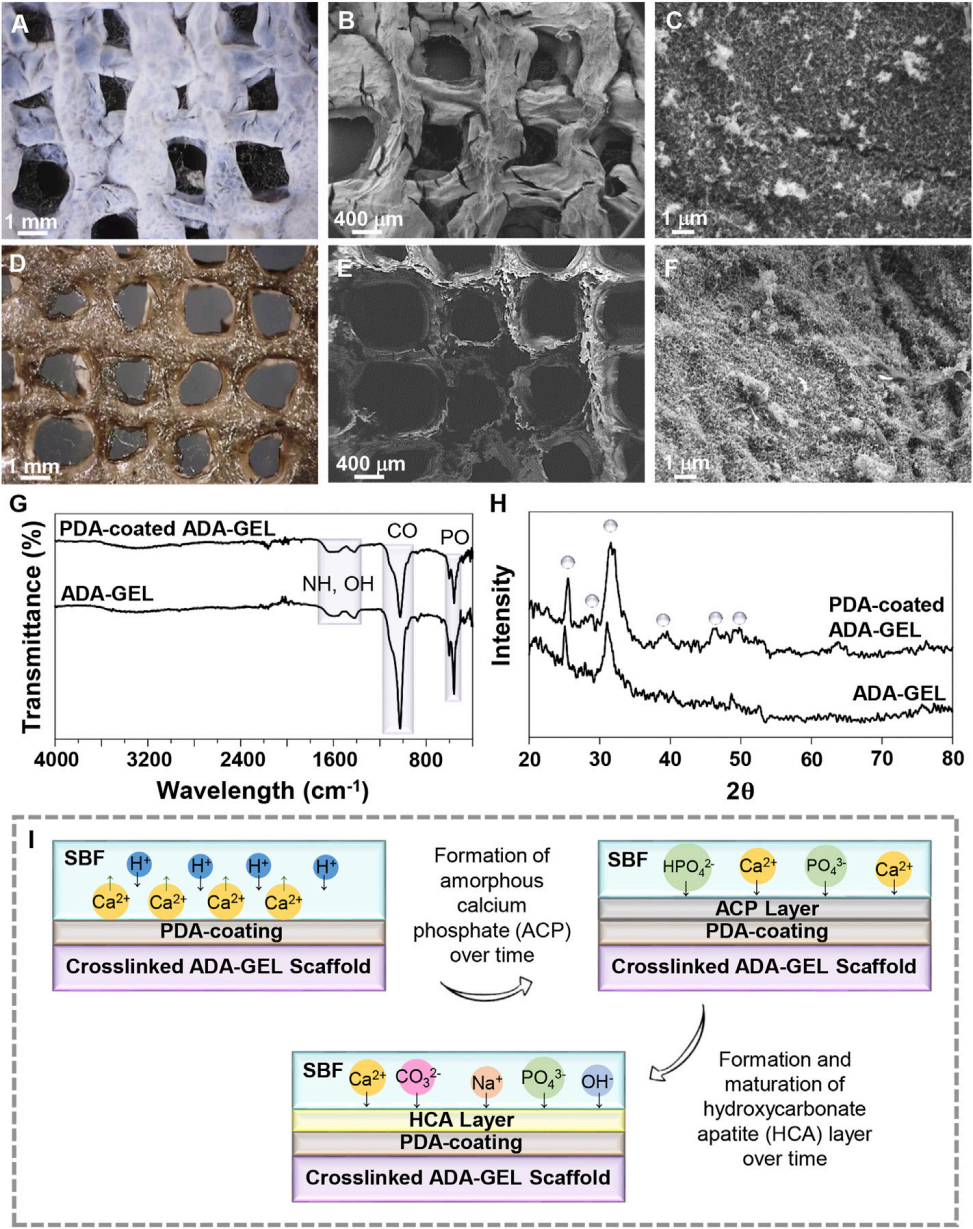
*In-vitro* bioactivity of the scaffolds. **(A,D)** Light microscope images and **(B,C,E,F)** FE-SEM images of **(A–C)** ADA-GEL and **(D–F)** PDA-coated ADA-GEL scaffolds after 18 days of immersion in the SBF solution. **(G)** FTIR spectrum and **(H)** XRD spectrum of ADA-GEL and PDA-coated ADA-GEL after biomineralization of HA-like layers. **(I)** Schematic illustration of the HA formation mechanism on scaffolds.

The ability to form an HA layer on the scaffolds’ surface in contact with physiological fluids is the sign of bioactivity for bone regeneration. According to Figures 5A–F, both the light microscope and FE-SEM images demonstrate that, while the calcium phosphate layer is locally condensed and aggregated on the surface of the uncoated ADA-GEL scaffold, it is uniformly distributed on the surface of the PDA-coated ADA-GEL scaffold after 18 days incubation in SBF solution. Crystals formed on the surface of the ADA-GEL scaffold are more irregular than those formed on the surface of the PDA-coated ADA-GEL scaffold. In preliminary studies, it has been demonstrated that PDA provides more active sites for the formation of HA layers (Yu et al., 2021). Ca^2+^ ions are also anticipated to have a synergetic effect with PDA during the formation of HA. Light microscopy and FE-SEM images of the scaffolds after the bioactivity test demonstrate that the PDA-decorated ADA-GEL scaffolds have improved structural integrity and calcium phosphate formation ability.

Infrared spectroscopy was used to examine the formation of the mineralized layer on ADA-GEL and PDA-functionalized ADA-GEL after immersion in the SBF solution. Figure 5G illustrates the FTIR spectra in both experimental groups after 18 days of immersion in the SBF solution. A peak at 582 cm^−1^ is associated with the P-O bonds. C-O vibrations, exhibited at 819 cm^−1^, are demonstrated for synthesized HA. The peak at 1,603 cm^−1^ is attributed to O-H stretching. The results of XRD analysis (Figure 5H) indicate deposition of a HA layer on ADA-GEL and PDA-coated ADA-GEL scaffolds. The characteristic peaks of HA at 2θ 25.26°, 28.87°, 31.60°, 39.65°, 45.62°, and 49.85° are assigned to crystallographic planes (002), (210), (211), (310), (222), and (213), respectively (Wei et al., 2011; Han et al., 2017). After mineralization of the HA layer on ADA-GEL scaffolds decorated with PDA, these peaks are evident, whereas they are less intense or have entirely disappeared on mineralized ADA-GEL scaffolds. In comparison with the uncoated ADA-GEL scaffolds, the PDA-coated scaffolds show sharper and more intense XRD peaks that may indicate higher crystallinity of precipitated HA.

A schematic illustration of the formation of HA layer is shown in Figure 5I. The release of hydrogen ions from the hydroxyl groups of the scaffold occurs upon immersion in SBF, leading to the sedimentation of acidic compounds such as hydrogen carbonate and hydrogen phosphate on the surface of the samples, which act to promote the formation of calcium phosphate (Ghorbani et al., 2019b). A thin layer of amorphous calcium phosphate is first formed on the scaffold. The ions then interact with the surface, resulting in the formation of a crystalline layer of HA. This is likely the result of the charge repulsion and numerous catecholamines present in PDA (Ryu et al., 2010; Ding et al., 2016). The hydrophilic nature of PDA also contributes to the absorption of calcium and phosphate ions from SBF solution (Ghorbani et al., 2019a).

### 3.6 Protein interaction

**FIGURE 6:**
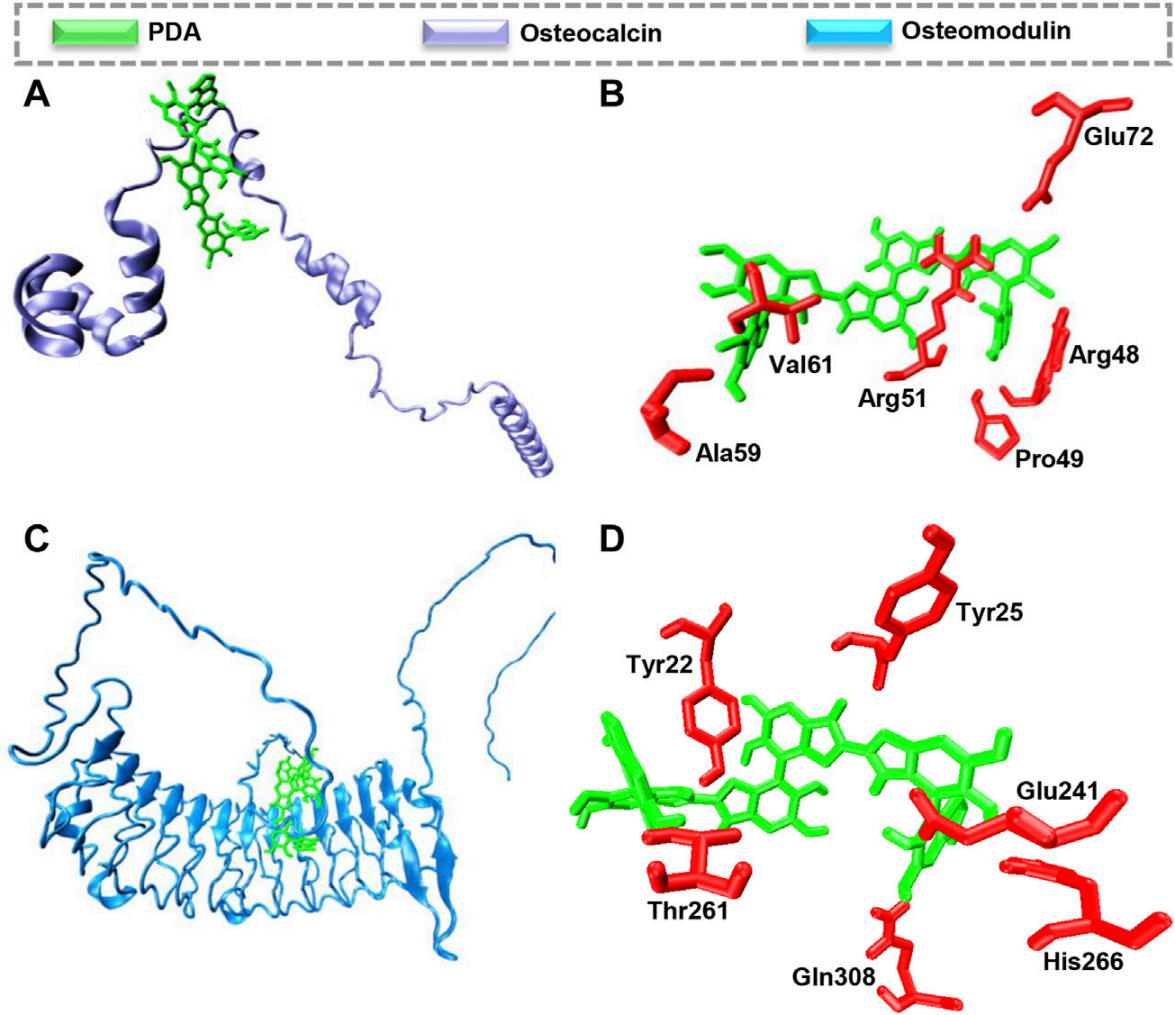
Molecular docking calculations of PDA interaction with osteocalcin and osteomodulin. The hydrogen bond is the driving force to form the proteins-PDA complex. **(A)** PDA binding to osteocalcin **(B)**, the best mode of PDA interaction with osteocalcin in the binding site, **(C)** PDA binding to osteomodulin **(D)** the best mode of PDA interaction with osteomodulin in the binding site.

Molecular docking calculations reveal that PDA is located on the surface of osteocalcin and osteomodulin, indicating proteins’ surfaces are prone to interact with PDA, as shown in Figures 6A,C. Docking analysis indicates that hydrogen bonds are the major driving force leading to the interaction between PDA and osteocalcin and osteomodulin. As indicated in Figures 6B,D the best mode of PDA binding to osteocalcin and osteomodulin has been determined based on pose clustering of docking calculations. PDA’s chemical structure and surface properties are the principal factors contributing to the formation of hydrogen bonds with proteins. Additionally, the obtained theoretical binding energy indicates that the PDA binding process to proteins is spontaneous. The binding energy values calculated by AutoDock Vina for PDA interaction with osteocalcin and osteomodulin are −35.95 kJ mol^−1^ and −46.39 kJ mol^−1^, respectively. Molecular docking calculations suggest that the surfaces of osteocalcin and osteomodulin are structurally adapted to PDA. Based on the findings, it appears that PDA surfaces and proteins possess structural complementarity. In other words, proteins and PDA generally exhibit favorable structural compatibility. Such structural adaptation will result in the development of a layer of proteins on the surface of PDA, thereby increasing the biocompatibility of scaffolds.

### 3.7 Cell-scaffold interactions

**FIGURE 7:**
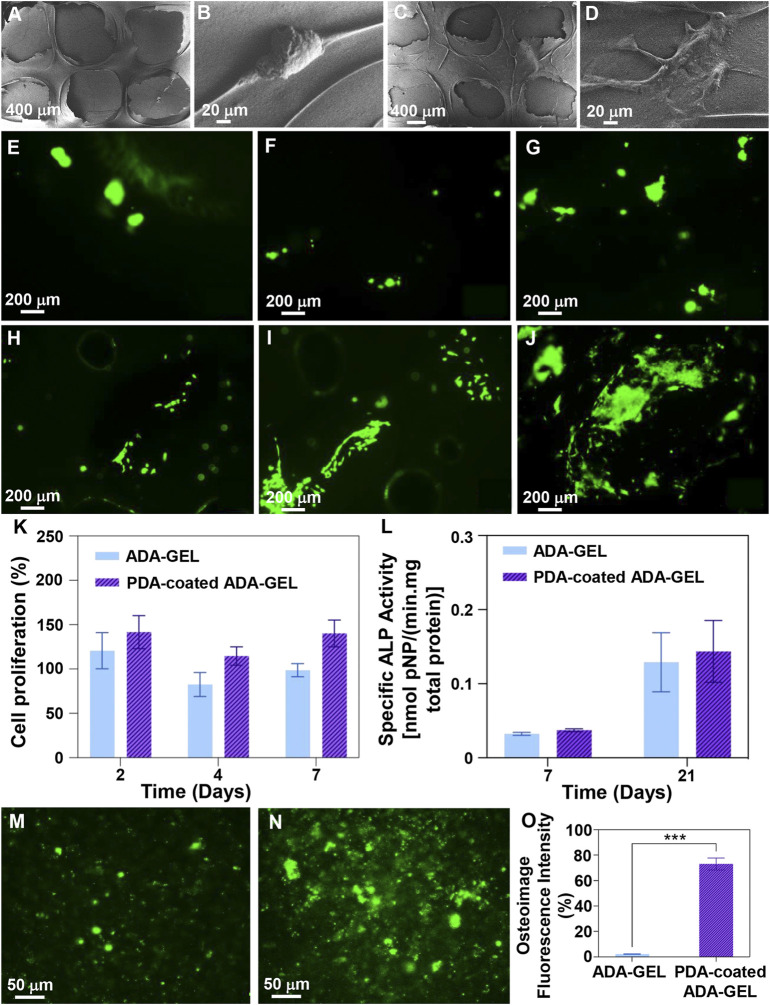
Cell-scaffolds interaction. **(A–D)** FE-SEM images of MG-63 cell adhesion on the surface of **(A,B)** ADA-GEL and **(C,D)** PDA-coated ADA-GEL scaffolds after 2 days culture. **(E–J)** MG-63 cell staining with calcein-AM after **(E,H)** 2, **(F,I)** 4, and **(G,J)** 7 days culture on **(E–G)** ADA-GEL and **(H–J)** PDA-coated ADA-GEL scaffolds, indicating cell viability. **(K)** Proliferation of MG-63 cells on the scaffolds as a function of incubation time. **(L)** ALP activity of MG-63 cells on the scaffolds after 7 and 21 days of culture. **(M–O)** Osteoimage mineralization of **(M)** ADA-GEL and **(N)** PDA-coated ADA-GEL scaffolds and **(O)** related quantitive results after 21 days cell culture. Differences are considered considerably significant (***p < 0.001).

This study examined the simple cell-scaffold interaction following the cultivation of MG-63 cells on scaffolds. Following a two-day culture, FE-SEM imaging (Figures 7A–D) demonstrated that polymeric substrates and designed 3D structures strongly support cell adhesion, indicating non-cytotoxicity of scaffolds and provision of cell anchorage sites. Besides, nutritionally-rich media, a reduced stress level, and adequate cell respiration are the other factors that may contribute to the development of this phenomenon (Liu et al., 2019). PDA decorated scaffolds however showed that MG-63 cells could adhered and spread well with increased filopodia and lamellipodia. Other investigations have indicated that in addition to hydrophobicity that arises from functionalization with PDA, abundant polar groups can enhance nutrient transport and interactivity with serum proteins can lead to introduction of cell anchorage sites, augmenting adsorption of endogenous fibronectin and integrin α5β1, and finally improving cell adhesion and growth (Shen et al., 2007; Wang et al., 2013; Liu et al., 2015). Parallel, Kao et al. (Kao et al., 2015) demonstrated that functionalized 3D printed poly (lactic acid) platforms by a mussel-inspired coating can be used to improving adhesion, proliferation, and differentiation of human adipose-derived stem cells (hADSCs). In addition, cell staining with calcein AM after 2, 4, and 7 days of culture (Figure 7E–I) confirmed that scaffolds can function as anchorage sites for cells, especially after functionalization with PDA, since clusters of living cells emerged after 4 days of culture and their intensity and homogeneity increased after 7 days. On the basis of proliferation assays (Figure 7K), PDA coated ADA-GEL scaffolds have shown higher proliferation than non-coated ADA-GEL scaffolds, which may be due to reduced protein denaturation by adjusting the surface energy and enhanced cell receptor interaction with PDA (Ding et al., 2016). Ku et al. (2010) showed that protein denaturation is related to surface energy, which affects protein conformation and cell adhesion. In conjunction with a lower wettability of the substrate, low surface energy results in protein denaturation due to exposure of the inner hydrophobic residues of the proteins, affecting adsorbate-cell interactions. After 2 days of incubation, cells began to proliferate well, while the number of viable cells decreased after 4 days. The value was, however, increased as a function of incubation time until 7 days. Proliferation assays may be influenced by swelling and degradation characteristics of scaffolds. As a result of the 10% degradation within the first 4 days, some cells were washed out or died due to stresses induced by the degradation. As time passes, the cells that were able to anchor well will grow and proliferate, resulting in an increase in the number of cells within 7 days. ALP activity (Figure 7L) has been analyzed 7 and 21 days after cellular culture as a non-collagenous biomarker for osteogenesis. Both experimental groups experienced an increase in ALP secretion after 21 days, as expected. Interestingly, PDA-coated scaffolds showed greater levels of ALP, indicating their potential for osteogenic activity. Furthermore, quantitative and qualitative osteoimage (Figure 7M−O) results showed that PDA-coated scaffolds can induce a higher level of mineralization when compared to neat ADA-GEL. This effect is likely the result of an improved bioactivity of the scaffolds (Section 3.5). The PDA surface’s ability to absorb bioactive molecules enhances cell differentiation and promotes an osteoinductive environment that accelerates bone regeneration (Lin and Fu, 2016). In general, the higher the level of ALP secreted following PDA surface modification, the greater the amount of calcium deposited and the greater the rate of mineralization (Ge et al., 2014).

## 4 Conclusion

ADA-GEL scaffolds were generated via an extrusion-based 3D printer and coated with mussel-derived adhesive protein (PDA) to facilitate bone regeneration. Aside from morphological, physicochemical, and mechanical evaluations, protein interaction modelling and *in vitro* performance evaluations were also conducted. In accordance with the obtained results, tubular-like pores were manufactured with 3D printing technology that allows for nutrient supply and ion exchange, as well as the stimulation of cell growth. Using PDA coatings on substrates, the mechanical performance and hydrophilicity of scaffolds were both improved, and degradation was more controlled. In addition, Calcium ions and PDA worked synergistically to increase biomimetic formation of HA layers. In Molecular Docking calculations, osteocalcin and osteomodulin were identified as potential targets for interaction with PDA, indicating that the preserve of PDA will contribute to osteoinduction. The functionalized scaffolds serve as a platform for adhesion and proliferation of MG-63 cells. Osteodifferentiation was demonstrated by an increase in ALP activity and osteoimage levels after PDA treatment. Overall, surface-modified scaffolds developed in this study exhibit encouraging potential as advanced biomaterials for bone TE.

## Data Availability

The original contributions presented in the study are included in the article/Supplementary Material, further inquiries can be directed to the corresponding authors.
